# Effect of Surface Ultrasonic Rolling Treatment on Rolling Contact Fatigue Life of D2 Wheel Steel

**DOI:** 10.3390/ma13235438

**Published:** 2020-11-29

**Authors:** Pengtao Liu, Zilong Lin, Chunpeng Liu, Xiujuan Zhao, Ruiming Ren

**Affiliations:** 1 School of Material Science and Engineering, Dalian JiaoTong University, Dalian 116028, China; liupptt@163.com (P.L.); qqprophet@126.com (Z.L.); liuchunpeng1991@sina.com (C.L.); zhaoxj@djtu.edu.cn (X.Z.); 2 Key Laboratory of Key Material of Rail Transit in Liaoning Province, Dalian JiaoTong University, Dalian 116028, China

**Keywords:** surface ultrasonic rolling treatment, D2 wheel steel, rolling contact fatigue life, surface microstructure

## Abstract

A GPM-30 fatigue machine was used to investigate the influence of surface ultrasonic rolling (SURT) on the rolling contact fatigue (RCF) life of D2 wheel steel. The experimental results present that the RCF life of the grinding processing sample is 4.1 × 10^5^ cycles. During the RCF process, the flaking of the fine grain layer and high surface roughness of the grinding processing sample cause the production of RCF cracks. When the samples are treated by SURT with 0.2 MPa and 0.4 MPa, the RCF life is 9.2 × 10^5^ cycles and 9.6 × 10^5^ cycles, respectively. After SURT, the surface roughness of the samples is reduced, and a certain thickness of gradient-plastic-deformation layer and a residual-compressive-stress layer are produced. These factors lead to the improvement of the RCF property. However, when the static pressure increases to 0.6 MPa during SURT, the RCF life of the sample is reduced during RCF testing. The micro-cracks, which are formed during SURT, become the crack source and cause the formation of RCF cracks, decreasing of the RCF life.

## 1. Introduction

During recent years, as the speed of high-speed trains has increased, the rolling contact fatigue (RCF) failure of wheel materials has become serious [1]. According to the results of Bevan et al. [2], the RCF of wheel materials is higher than other failure forms. During the operation process of high-speed trains, the wheel tread is subjected to cyclic stress. The cyclic stress results in local permanent cumulative damage. When the trains run a certain number of cycles, the wheel tread will produce severe plastic deformation causing ratcheting failure [3]. The RCF of wheel materials seriously affects the safety of train operation [4,5].

The process of RCF damage is very complicated. Some factors can change the fatigue performance of wheel steel. The surface defect of wheel materials can cause the stress concentration to decrease the fatigue resistance. According to the result of Kapoor et al. [6], the value of contact stress at the rough peaks is high. It is about 8 times higher than that of smooth surface. White etching layer, as the surface local defect, also can cause fatigue failure [7]. Many researchers have also studied the influence of lubricating fluid (water and oil) on RCF performance. The lubricating fluid enters the crack after the crack is generated, and the crack propagation is accelerated due to the influence of the lubricating fluid [8,9]. The RCF cracks of wheel and rail steel tend to initiate on the surface. Therefore, removing surface defects of wheel and rail steel is a significant way to improve fatigue life of wheel and rail steel. The grinding processing is a conventional method to decrease the surface defects of wheel and rail materials. When wheel/rail materials are grinded, a certain thickness of grinding-fine-grain layer is formed on the surface inevitably [10]. Additionally, the wheel and rail surface form obvious grinding marks, which can cause stress concentration. The grinding fine layer and grinding marks will affect the fatigue life of wheel and rail materials [11]. Surface ultrasonic rolling technology is a novel surface strengthening technology which can improve the surface mechanical property of materials and reduce the surface defect of materials. Surface ultrasonic rolling (SURT) can enhance surface hardness, decrease surface roughness, and lead to the formation of surface residual compressive stress. The variation of material surface property after SURT can change the fatigue life [12,13].

To study the influence of SURT on RCF performance of D2 wheel steel, two kinds of sample were prepared. One sample was the grinding processing sample (the last machining operation was grinding). Another sample was treated by SURT with different loads. The surface morphology, surface hardness, and fatigue cracks of two kinds of sample were studied. The mechanism of RCF failure of two kinds of samples is further discussed.

## 2. Experimental Methods

**Figure 1:**
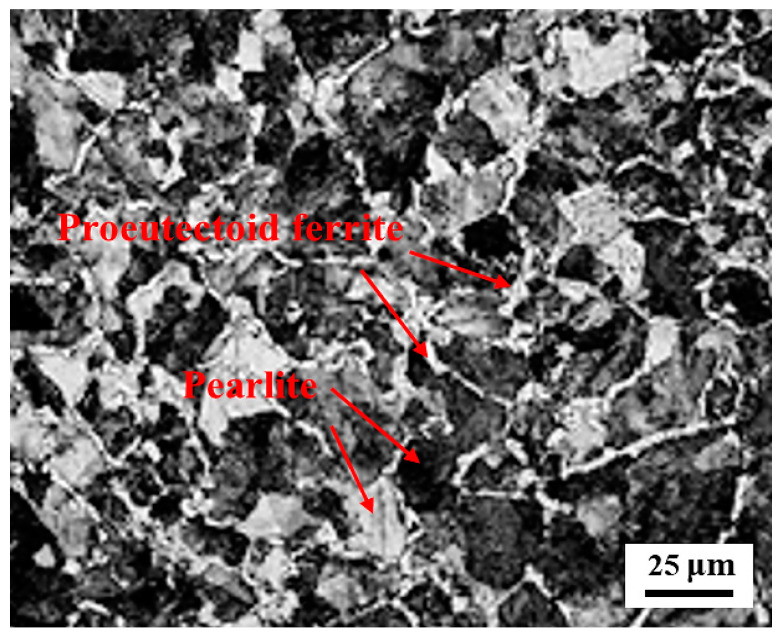
The original microstructure of D2 wheel steel.

**Figure 2:**
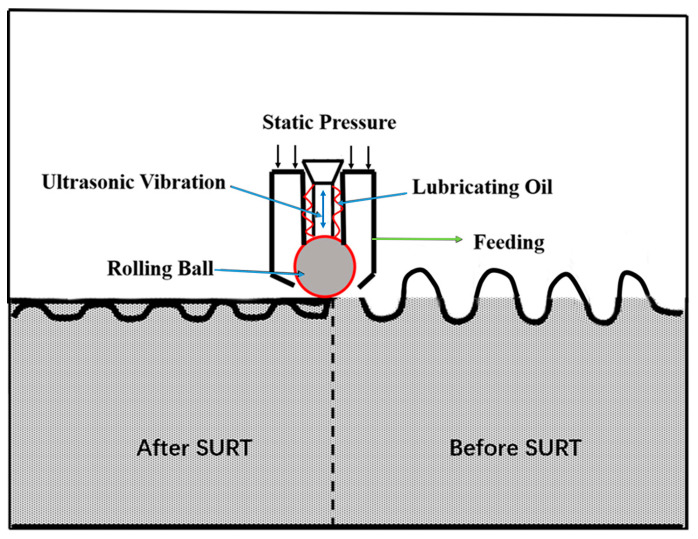
The schematic diagram of surface ultrasonic rolling machine [14].

**Figure 3:**
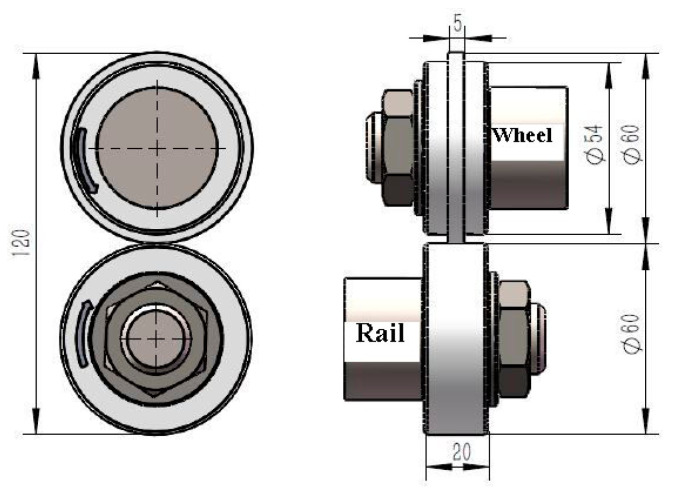
Sample dimensions and contact mode of wheel and rail samples (unit: mm) [15].

In this work, the test materials were D2 wheel steel and U71Mn rail steel. Table 1 displays the chemical compositions of the test samples. The original microstructure of the D2 wheel sample was lamellar pearlite and proeutectoid ferrite, as shown in Figure 1. The yield strength and tensile strength of the D2 wheel steel were 615 MPa and 955 MPa, respectively. The wheel sample had an original hardness of about 290 HV. The yield strength and tensile strength of the U71Mn rail steel were 880 MPa and 900 MPa, respectively, and the original hardness of the rail sample was about 330 HV. The grinding processing parameters were that grinding speed was 30 m/s, the speed of the sample was 10 m/min, the radial feed was 0.01 m, and the cooling mode was dry grinding. The H^+^B6063 surface ultrasonic rolling machine (Huayun, Jinan, China) was used to treat the surface of the wheel samples, as shown in Figure 2. The static pressure was 0.2, 0.4, and 0.6 MPa. The rolling speed was 70 r/min; the feed was 0.05 mm/r. The RCF tests were conducted by the GPM-30 fatigue tester (Yihua, Jinan, China). Figure 3 displays the samples’ dimensions and the contact mode of wheel and rail samples. For the RCF test, test contact stress was selected at 1450 MPa according to the S-N curve of D2 wheel steel [11]. The rolling speed was 1440 r/min to simulate the rolling speed of 250 km/h. The creep ratio was 0.5. No. 20 engine oil was used to lubricate the contact surface. When the surface spalling was larger than 3 mm^2^ the sample was fatigue failure.

After SURT and RCF tests, the surface microstructure and surface morphology of the samples were observed on a scanning electron microscope (FEG-SEM, Carl Zeiss, Jena, Germany). The surface roughness of the samples was measured by a Leica three-dimensional microscope (Leica Microsystems, Heidelberg, Germany). The surface hardness was obtained by an FM-700 hardness tester (Future-Tech Corporation, Kawasaki, Japan). The load was 0.245 N for 15 s. The surface residual stress of the wheel samples was obtained by an i-XRD X-ray residual stress tester (Proto Company, Windsor, ON, Canada) before and after SURT.

## 3. Results

### 3.1. Rolling Contact Fatigue Life

**Figure 4:**
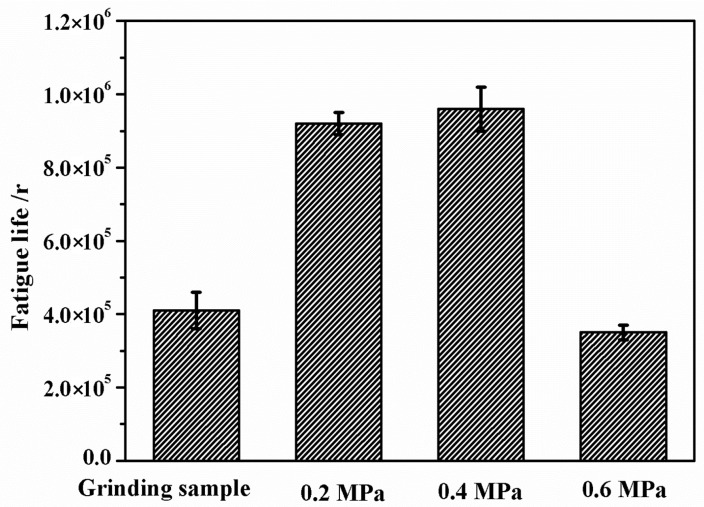
Variation of rolling contact fatigue (RCF) life of the grinding processing sample and the samples after surface ultrasonic rolling (SURT) with different static pressures.

Figure 4 presents the rolling contact fatigue (RCF) life of the grinding processing sample and the sample after SURT with different static pressures. Six repeated RCF tests were carried out at each SURT parameter. The RCF life of the grinding processing sample was about 4.1 × 105 cycles. The RCF life of the sample after SURT with 0.2 MPa was about 9.2 × 105 cycles. When the static pressure increased to 0.4 MPa, the RCF life of the sample after SURT was about 9.6 × 105 cycles. However, when the static pressure reached to 0.6 MPa, the RCF life of the sample reduced to about 3.5 × 105 cycles. The RCF life of the samples after SURT with 0.2 MPa and 0.4 MPa was 2.2 times and 2.3 times that of the grinding processing sample, respectively. However, the RCF life of the samples after SURT with 0.6 MPa was 0.8 times that of the grinding processing sample. Therefore, the surface morphology and microstructure changes of the samples after SURT had a significant influence on the D2 wheel samples.

### 3.2. Surface Morphology after SURT

**Figure 5:**
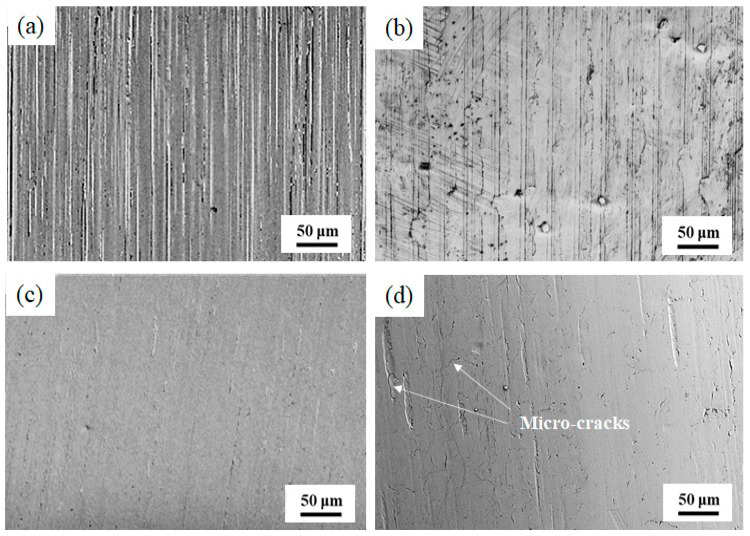
The surface micro-morphology of the grinding processing sample and the sample after SURT with different static pressures. (**a**) The grinding processing sample; (**b**) the sample after SURT with 0.2 MPa; (**c**) the sample after SURT with 0.4 MPa; and (**d**) the sample after SURT with 0.6 MPa.

**Figure 6:**
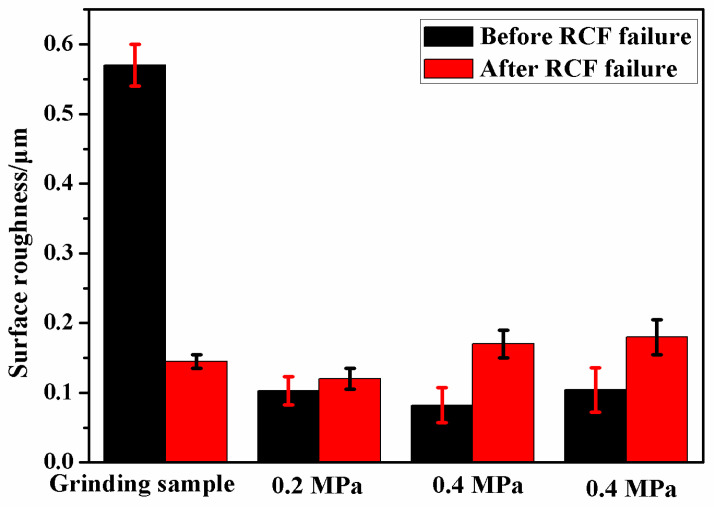
The surface roughness of the grinding processing sample and the sample after SURT with different static pressures before and after RCF.

Figure 5 presents surface micro-morphology of the grinding processing sample and the samples after SURT with different static pressures. After grinding processing, grinding marks were formed on the sample surface and obvious peaks and valleys were generated on the sample surface (Figure 5a). The surface roughness of the sample after grinding processing was also relatively large; it was 0.57 μm, as shown in Figure 6. When the sample was treated by SURT with 0.2 MPa, the sample surface became smooth, and the difference between valley and peak on the sample surface was reduced (Figure 5b). The surface roughness of the sample decreased significantly, to about 0.103 μm. When static pressure increased to 0.4 MPa, the surface of the sample became obviously smooth. Moreover, there were no peaks and valleys at the sample surface, as shown in Figure 5c. The surface roughness of the sample was further reduced to 0.082 μm. However, when the static pressure increased to 0.6 MPa, obvious micro-cracks were produced at sample surface after SURT, and the surface roughness increased to 0.104 μm (Figure 6). The micro-cracks on the sample surface became a source of RCF cracks, accelerating fatigue failure [16]. Therefore, when the sample underwent SURT with 0.6 MPa, the RCF life of the sample was decreased. After RCF failure, the surface roughness of samples was measured. It was found that the roughness of the grinding processing sample decreased significantly, to about 0.145 μm, while the roughness of the samples after SURT slightly increased after RCF failure. To study the reasons that SURT enhanced the RCF life, the microstructure, hardness, and residual stress of the grinding processing samples and the samples after SURT with 0.4 MPa were analyzed systematically.

### 3.3. Surface Microstructure

**Figure 7:**
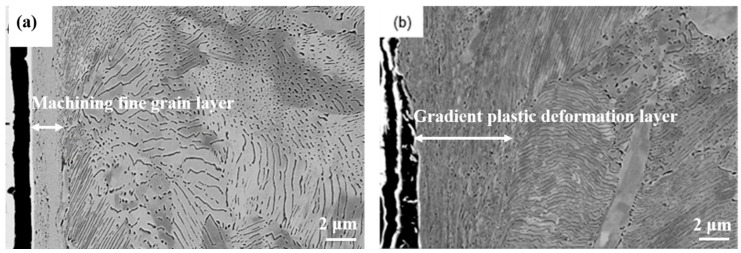
The SEM micrographs: (**a**) the grinding processing sample and (**b**) the sample after SURT with 0.4 MPa.

Figure 7 displays the SEM micrographs of the microstructure evolution of the grinding processing sample and the sample after SURT with 0.4 MPa. After grinding treatment, a fine grain layer with a thickness of approximately 1 μm was formed at the grinding processing sample, as shown in Figure 7a. During the grinding process, the contact condition of the sample surface and the grinding tool was slide wear, and the temperature at the sample surface was increased. The sample surface underwent significant plastic deformation. Thus, a certain thickness of fine grain layer was formed after the grinding processing. The fine grain layer exhibited high hardness and poor plasticity. Therefore, the plastic deformation between fine grain layer and core microstructure was inconsistent. During the RCF process, micro-cracks were formed at the interface of the fine grain layer and the core microstructure. After SURT with 0.4 MPa, a gradient plastic deformation layer with a thickness of about 10 μm was produced at the sample surface, as displayed in Figure 7b. At 0~2 μm away from the surface, an obvious plastic deformation layer was formed. The surface ferrite grains were obviously refined. Cementite platelets in the pearlite were fractured and dissolved. At the depth of 2~8 μm below contact surface, the cementite in pearlite was lamellar, but the ferrite grains were obviously deformed. After 8 μm from the surface, there was no plastic deformation in pearlite, and the cementite was still lamellar. However, fine grains were formed in the proeutectoid ferrite.

**Figure 8:**
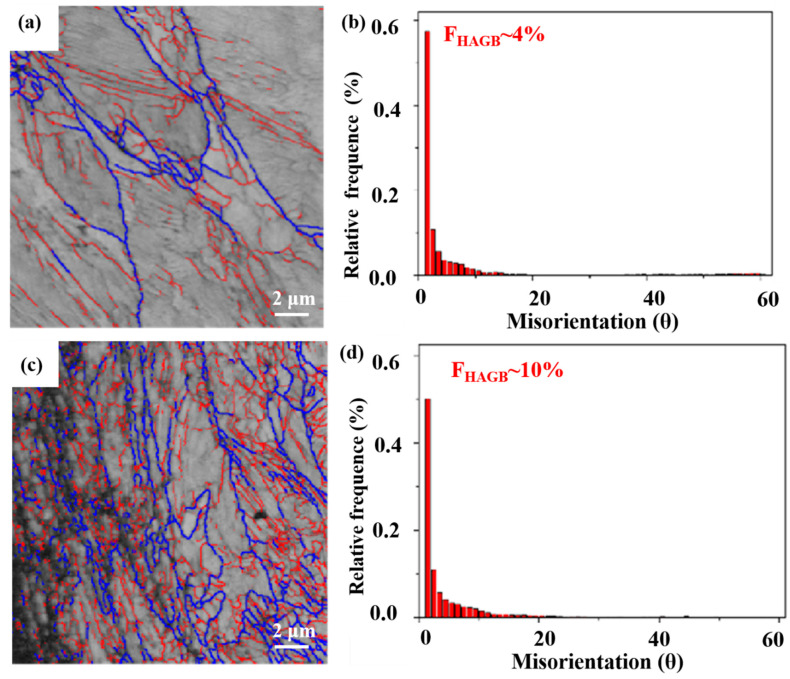
The EBSD micrographs: (**a**,**b**) the grinding processing sample and (**c**,**d**) the sample after SURT with 0.4 MPa. (Red lines (low-angle grain boundary): 2° < θ < 10°; blue lines (high-angle grain boundary): θ > 10°.)

EBSD was used to systematically analyze the variation of ferrite grains of the grinding processing sample and the sample after SURT with 0.4 MPa, as shown in Figure 8. The size of ferrite grains of the grinding processing sample was relatively large and the fraction of high-angle boundary grain (HABG) was only 4% (Figure 8a,b). Figure 8c,d show the EBSD micrographs of the sample after SURT with 0.4 MPa. After SURT, the ferrite grains were significantly refined and the fraction of HAGB increased to 10%. According to the result of Izotov et al. [17], the mechanism of the refinement of ferrite grains and increase of HAGB was that with plastic strain increasing, the dislocation density was increased. High dislocation density changes to sub-boundaries. Finally, the sub-boundaries changed into the HAGB.

**Figure 9:**
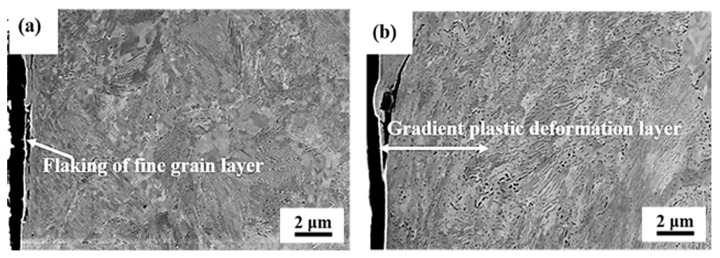
The SEM micrographs of the microstructural evolution of samples after RCF failure: (**a**) the grinding processing sample and (**b**) the sample after SURT with 0.4 MPa.

Figure 9 shows the change of microstructure of the grinding processing sample and the sample after SURT with 0.4 MPa after RCF failure. The fine grain layer on the grinding processing sample surface was flaked due to the inconsistent plastic deformation between the fine grain layer and the core microstructure (Figure 9a). However, after RCF failure, the plastic deformation layer of the sample after SURT with 0.4 MPa was not flaked, as shown in Figure 9b, although the RCF crack was formed. The reason for this is that the deformation layer of the sample after SURT with 0.4 MPa changes in gradient from the surface to the undeformed zone. The plastic deformation layer after SURT with 0.4 MPa was not peeled off after RCF failure.

### 3.4. Surface Hardness and Residual Stress

**Figure 10:**
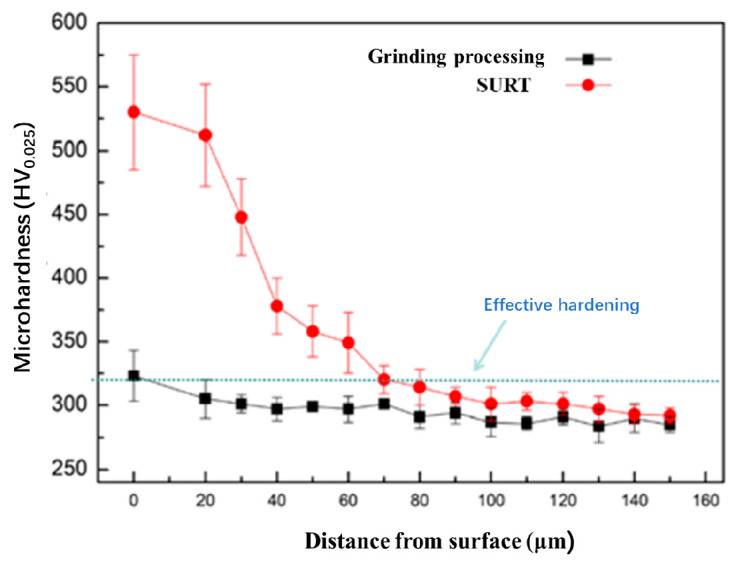
The variation of hardness of the grinding processing sample and the sample after SURT with 0.4 MPa.

Figure 10 shows the variation of hardness of the grinding processing sample and the sample after SURT with 0.4 MPa from surface to undeformed zone. The contact surface hardness of the grinding processing sample was 340 HV, which was higher than that of original hardness. This was because a fine grain layer was formed on the surface of the grinding processing sample. With the increase of distance from the surface, the hardness dropped rapidly. At 20 μm away from the surface, the sample reached the original hardness, which was about 300 HV. After SURT, the hardness of the sample increased significantly, and the surface hardness reached 520 HV. As the distance from the surface increased, the hardness of the samples decreased gradually. At 90 μm away from the surface, the hardness of the sample after SURT was same as that of the original hardness.

**Figure 11:**
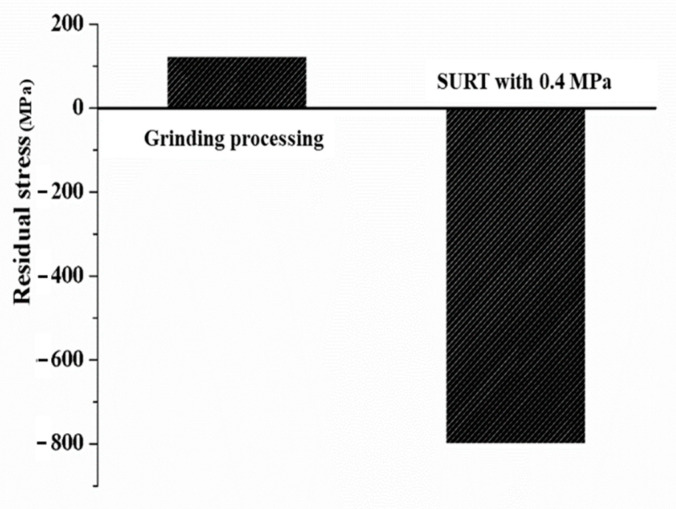
The variation of the surface residual stress of the grinding processing sample and the sample after SURT with 0.4 MPa.

The variation of surface residual stress of the grinding processing sample and the sample after SURT with 0.4 MPa is shown in Figure 11. After grinding processing, the sample surface formed residual tensile stress, which was about 123 MPa. However, after SURT, the residual stress of the sample transformed from residual tensile stress to residual compressive stress. Its value was about −800 MPa.

### 3.5. Rolling Contact Fatigue Cracks

**Figure 12:**
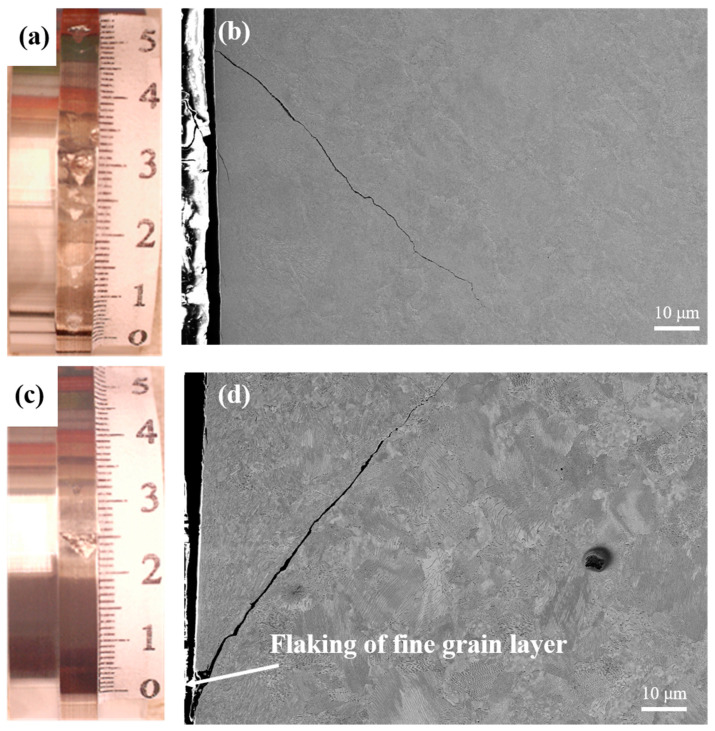
Surface morphology and RCF cracks: (**a**,**b**) the grinding processing sample and (**c**,**d**) the sample after SURT with 0.4 MPa.

Figure 12 shows the surface macro-morphology of the grinding processing sample and the samples after SURT with 0.4 MPa after RCF failure. After RCF failure, two kinds of samples surface produced bulking flaking. However, the fatigue crack initiation process of the grinding sample and the sample after SURT with 0.4 MPa was different. The RCF cracks of the sample after SURT with 0.4 MPa were initiated at the surface and propagated along the direction of about 45° with the surface. The plastic deformation layer formed at the surface after SURT was able to delay the initiation of RCF cracks [18]. During the RCF process, for the grinding processing sample, first, the fine grain layer on the surface was peeled off and the original microstructure was exposed at the sample surface. As the cycle increased, the surface original microstructure produced plastic deformation, and dislocation density increased. Two factors were able to accelerate RCF cracks’ initiation [19].

**Figure 13:**
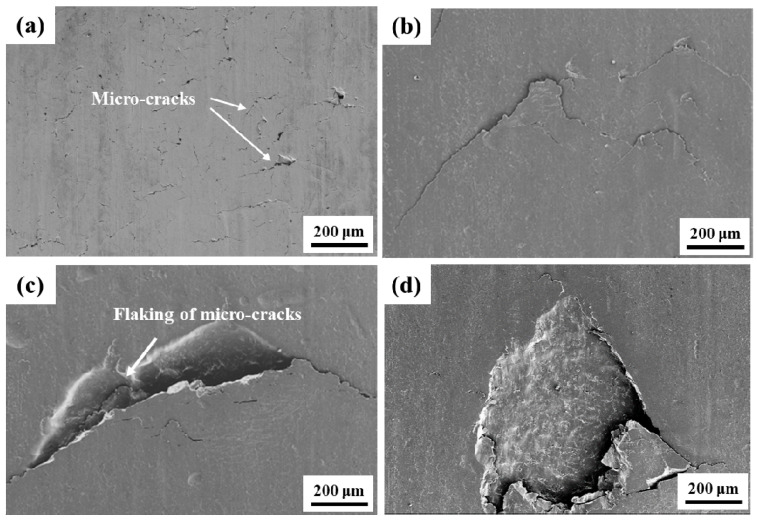
The propagation process of RCF cracks of the grinding processing sample. (**a**) Surface micro-cracks; (**b**) micro-crack propagation; (**c**) RCF crack propagation; (**d**) the surface large spalling.

**Figure 14:**
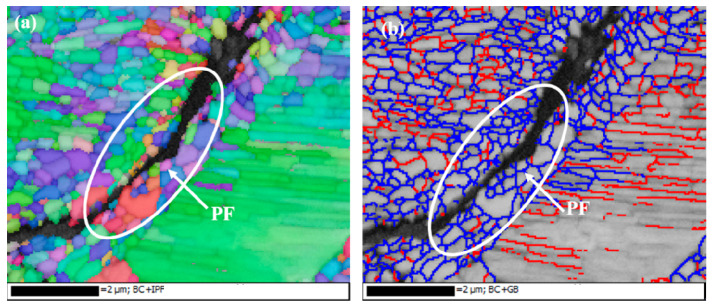
The EBSD micrographs of propagation of RCF cracks in proeutectoid ferrite (**a**) Inverse pole ﬁgure; (**b**) Grain misorientation figure (red lines: 2° < θ < 10°; bule lines: θ > 10°; “PF” is an abbreviation of proeutectoid ferrite).

**Figure 15:**
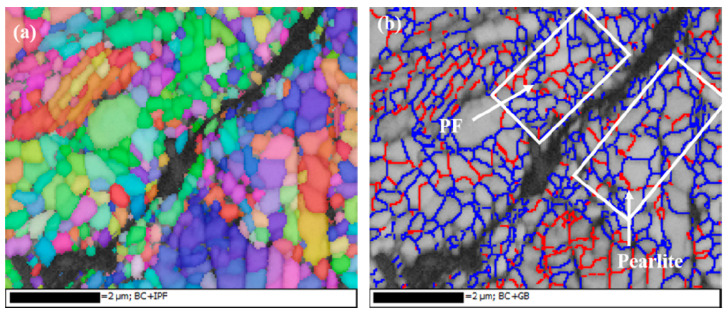
The EBSD micrographs of propagation of RCF cracks at the interface between proeutectoid ferrite and pearlite (**a**) Inverse pole ﬁgure; (**b**) Grain misorientation figure (red lines: 2° < θ < 10°; blue lines: θ > 10°; “PF” is an abbreviation of proeutectoid ferrite).

After RCF cracks’ initiation, the RCF cracks propagation process of the grinding processing sample and the sample after SURT with 0.4 MPa was the same, as shown in Figure 13. During the RCF process, most of the fatigue cracks initiated at the sample surface. The plastic strain was increased due to the cyclic stress and friction force during the RCF process. When the increase of plastic strain exceeded the ductility limit of the material, micro-cracks were formed on the surface, as shown in Figure 13a. As the cycle increased, the micro-cracks on the sample surface gradually propagated, as shown in Figure 13b. As the surface micro-cracks grew to a certain extent, the surface micro-cracks were peeled off, as shown in Figure 13c. When the surface micro-cracks were flaked, the lubricating oil entered the micro-crack. It was able to accelerate the RCF cracks’ formation and eventually lead to fatigue failure (Figure 13d). In order to study the relationship between growth of RCF cracks and microstructure during the RCF process, EBSD was used to systematically analyze the microstructure around RCF cracks, as shown in Figure 14. RCF cracks mainly propagated inside the proeutectoid ferrite (Figure 14a,b), because the proeutectoid ferrite was a soft phase. During the RCF process, the proeutectoid ferrite quickly reached the fatigue limit and led to the formation of RCF cracks [20]. As shown in Figure 15a,b, the RCF cracks grew along the interface between proeutectoid ferrite and pearlite. The reason for this is that the plastic deformation between pearlite and proeutectoid ferrite was not coordinated during the RCF process [21].

## 4. Discussion

**Figure 16:**
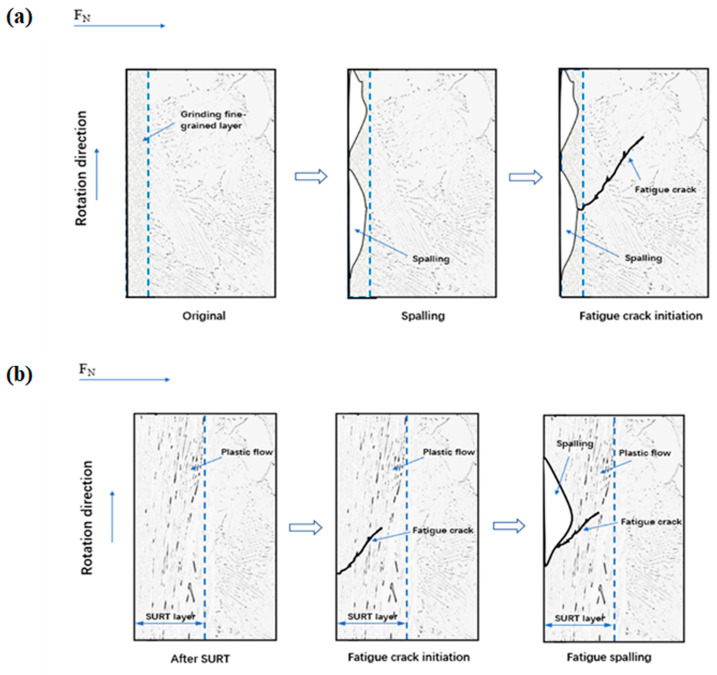
The schematic diagram of the formation process of RCF cracks: (**a**) the grinding processing sample and (**b**) the sample after SURT.

Figure 16a shows the formation process of RCF cracks of the grinding processing sample. After grinding, a certain thickness of grinding fine-grained layer was formed after grinding processing. During the RCF process, with the increase of cycles, the grinding fine-grain layer was flaked. Additionally, the original microstructure of the sample was exposed. As the cycles further increased, RCF cracks were formed in the original microstructure. The flaking of fine grain layer was able to accelerate RCF failure. After SURT, a gradient-plastic-deformation layer was produced on the sample surface. The gradient-plastic-deformation layer was not flaked after RCF failure, as shown in Figure 16b. The EBSD results show that after SURT, a fine ferrite grain layer was formed on the sample surface. Fine ferrite grain boundaries were able to hinder RCF cracks’ initiation. The reason for this is that grain boundary can prevent dislocation movement [22]. Therefore, the fatigue life of the samples can be improved after SURT. After SURT, high-density dislocations were generated, which can increase the yield strength and flow stress of the sample. The dislocations entanglement after SURT can hinder the dislocation slip on the sample surface during RCF process. Therefore, the refinement of ferrite grains of the sample after SURT can hinder the initiation of RCF cracks and improve the fatigue life. The sample surface generated the residual tensile stress layer after grinding processing, which can accelerate the formation of RCF cracks. The residual compressive stress layer, which is formed on the surface of the sample after SURT, can hinder the initiation of RCF cracks. Some published research results also indicate that residual compressive stress can inhibit the initiation and propagation of fatigue cracks [23,24,25,26,27,28].

## 5. Conclusions

A study on influence of SURT on the RCF life of D2 wheel steel was conducted using a GPM-30 fatigue tester. The surface morphology, surface microstructure, surface residual stress, and microhardness of the grinding processing sample and SURT samples were investigated. The following conclusions were obtained:

1. For the grinding processing sample, the interface between the fine ferrite grain layer and the core microstructure causes the flaking of the fine grain layer. The flaking of the fine grain layer accelerates the formation of RCF cracks.
2. SURT can lead to a decrease of the surface roughness, a gradient hardening layer, and a residual compressive layer at the sample surface to enhance the RCF life before micro-cracks are produced at the sample surface during SURT process.
3. When the micro-crack is formed during SURT, it becomes a source of the RCF crack, accelerating RCF crack initiation and causing an obvious reduction of RCF life.

## Figures and Tables

**Table 1:** Chemical compositions of the samples (wt %).

Samples	C	Si	Mn	S	P
D2	0.50–0.56	0.90–1.10	0.90–1.10	≤0.010	≤0.015
U71Mn	0.65–0.77	0.15–0.35	1.00–1.40	≤0.03	≤0.03
